# The Effect of Glycemic Control on Cardiovascular Disease Progression in Adults With Early-Onset Type 2 Diabetes: A Longitudinal Cohort Analysis

**DOI:** 10.7759/cureus.75058

**Published:** 2024-12-03

**Authors:** Amna Gilani, Khalid Umar, Fatima Gilani, Muhammad Ahmad, Mahnoor S Abbasi, Muhammad Yaseen, Muhammad Zeeshan, Naqeeb Ullah, Aiman Waseem, Fatima Batool, Sundas Safdar

**Affiliations:** 1 Pediatrics, Ayub Teaching Hospital, Abbottabad, PAK; 2 General Medicine, Ayub Teaching Hospital, Abbottabad, PAK; 3 Medicine and Surgery, Ayub Teaching Hospital, Abbottabad, PAK; 4 Anesthesia and Intensive Care, Chaudhary Pervaiz Elahi Institute of Cardiology Multan, Multan, PAK; 5 Medicine, Ayub Teaching Hospital, Abbottabad, PAK; 6 Internal Medicine, Ayub Teaching Hospital, Abbottabad, PAK; 7 Internal Medicine, Lady Reading Hospital Peshawar, Peshawar, PAK; 8 Anesthesia, Ayub Teaching Hospital, Abbottabad, PAK; 9 Medical Acute Unit, St. Vincent’s Private Hospital, Dublin, IRL; 10 Medicine, Khyber Medical University, Peshawar, PAK; 11 Diagnostic Radiology, Lady Reading Hospital Peshawar, Peshawar, PAK

**Keywords:** blood pressure, cardiovascular disease, cohort research, early-onset diabetes, glycemic management, lipids, type 2 diabetes mellitus

## Abstract

Introduction

Rising prevalence rates of type 2 diabetes mellitus (T2DM), particularly in younger populations, have made early-onset T2DM (diagnosed before age 40) an increasingly significant health concern. Early-onset T2DM is often associated with more rapid progression and increased complications, including cardiovascular disease (CVD). However, its specific impact on cardiovascular outcomes remains inadequately understood, particularly compared to T2DM in older populations. This study aimed to assess how glycemic management affects the course of CVD in individuals with early-onset T2DM.

Methodology

During the six months between December 2, 2023, and August 5, 2024, a longitudinal cohort study was carried out at Ayub Teaching Hospital in Abbottabad. In total, 470 adults with early-onset T2DM were included in the study cohort after applying exclusion criteria. Participants were classified into two groups based on glycated hemoglobin (HbA1c) values: those with HbA1c ≤ 7% and those with HbA1c > 7%. Using SPSS version 27 (IBM Corp., Armonk, NY, US), data were analyzed as follows: Baseline characteristics were compiled using descriptive statistics, with mean and standard deviation for continuous variables and frequencies for categorical variables. Time to cardiovascular events relative to glycemic control levels was assessed using Kaplan-Meier survival analysis. To examine the relationship between HbA1c levels and the risk of CVD development, Cox proportional hazards models were employed, adjusting for potential confounders such as age, sex, diabetes duration, BMI, and lipid profile. Differences in continuous variables were analyzed using two-sample t-tests, with p-values < 0.05 considered statistically significant.

Results

This study assessed the impact of glycemic management on CVD progression in individuals with early-onset T2DM. A total of 470 participants were included, with those having HbA1c > 7% showing a significantly higher risk for cardiovascular events (hazard ratio: 1.88, 95% CI: 1.25-2.85, p < 0.01). Participants with higher HbA1c levels also exhibited worse lipid profiles, including elevated LDL cholesterol (130.4 mg/dL vs. 115.2 mg/dL, p < 0.01) and triglycerides (178.6 mg/dL vs. 150.7 mg/dL, p < 0.01), along with increased blood pressure. These findings highlight the critical role of glycemic control in CVD risk, particularly in younger populations with early-onset T2DM.

Conclusion

Maintaining HbA1c levels below 7% is crucial for reducing cardiovascular risk in individuals with early-onset T2DM. This study highlights the importance of comprehensive management strategies that focus on glycemic control, lipid regulation, and blood pressure management. These strategies should be implemented through evidence-based interventions, such as lifestyle modifications (e.g., dietary changes, physical activity), pharmacological treatments (e.g., metformin, statins, antihypertensive medications), and regular monitoring to improve cardiovascular outcomes. While the findings are based on a cohort from Ayub Teaching Hospital, they are likely relevant to similar populations with early-onset T2DM, though generalizability to other regions or healthcare settings should be considered with caution.

## Introduction

Prevalence

Rising prevalence rates linked to ever more sedentary lifestyles, urbanization, and dietary changes have made type 2 diabetes mellitus (T2DM) a serious worldwide health concern [1]. Originally a condition of older persons, T2DM is now diagnosed more often in younger groups, leading to what is known as early-onset T2DM, defined as onset before age 40 [2]. This change in the age of onset not only exposes people to the disease for a longer period but also usually corresponds with a more aggressive clinical course, marked by rapid progression to serious complications [3]. Among these problems, cardiovascular disease (CVD) is the main cause of morbidity and death in T2DM patients, contributing to both personal and system costs [4].

Pathophysiology

Hyperglycemia-induced mechanisms, including endothelial dysfunction, chronic inflammation, oxidative stress, and lipid deregulation, drive the well-documented link between T2DM and CVD. These mechanisms accelerate atherosclerosis and other cardiovascular diseases, significantly increasing lifetime risk, particularly for younger individuals with early-onset T2DM [5]. A major focus of diabetes care is glycemic control, and strong evidence supports its role in lowering the incidence of microvascular complications, such as retinopathy, nephropathy, and neuropathy [6]. However, while the benefits of strict glycemic control on these microvascular complications are well-established, the effects on macrovascular outcomes, particularly cardiovascular events, remain less clear-cut [7,8].

Clinical outcomes

Individuals with early-onset T2DM may experience different cardiovascular outcomes compared to those diagnosed later in life, possibly due to a longer exposure to hyperglycemia and other metabolic disturbances. This increased duration of disease could contribute to a more aggressive disease trajectory and a higher risk of cardiovascular events [9,10]. Recent studies have shown that early-onset T2DM is linked with a more rapid progression of CVD, but the precise impact of glycemic control on these cardiovascular outcomes remains unclear. Despite extensive research on glycemic control and microvascular complications, there is a lack of data specifically addressing how glycemic management influences cardiovascular events in this high-risk cohort. While new findings suggest that early-onset T2DM carries an elevated risk for CVD, current diabetes care guidelines do not adequately differentiate between early- and later-onset T2DM in terms of treatment strategies [5,11].

Study aims

The purpose of this study is to investigate how glycemic management affects the development of cardiovascular disease in adults with early-onset T2DM. Through a longitudinal cohort analysis, this project will assess whether maintaining ideal glycemic control, as measured by glycated hemoglobin (HbA1c) levels, influences the occurrence and severity of cardiovascular events over time. The results of this study will offer valuable insights into how consistent glycemic control can potentially reduce long-term cardiovascular risks in this high-risk population. Given the lack of data on cardiovascular outcomes in early-onset T2DM patients, this research aims to fill the knowledge gap and inform future treatment strategies that could improve patient outcomes, particularly in addressing cardiovascular complications.

## Materials and methods

Study design and setting

This longitudinal cohort study was conducted at Ayub Teaching Hospital, Abbottabad, Pakistan, from December 2, 2023, to August 5, 2024. The primary objective was to evaluate the relationship between glycemic control and cardiovascular outcomes in individuals with early-onset T2DM. Both incident cardiovascular events (e.g., myocardial infarction, stroke) and the progression of subclinical markers (e.g., carotid intima-media thickness and worsening hypertension) were studied. 

Ethical considerations

The study was reviewed and approved by the Ethics Committee of Ayub Teaching Hospital, Abbottabad, under approval number ATH/EC-10/17(2). All procedures were conducted in accordance with the Declaration of Helsinki. Written informed consent was obtained from all participants prior to their enrollment in the study. The confidentiality of participant data was maintained throughout the research, with robust safeguards to protect personal information.

Sample size calculation

The sample size was calculated based on an anticipated incidence of cardiovascular events in early-onset T2DM patients, considering an effect size of 0.3 (based on prior studies examining CVD progression in T2DM populations) [12]. Using a two-sided alpha of 0.05 and a statistical power of 80%, the required sample size was determined to be 470 participants. To account for potential dropouts, a 10% adjustment was made, bringing the total target sample size to approximately 520 participants. A total of 500 participants were initially recruited. However, after screening for eligibility and following the study protocol, 30 participants were excluded for the following reasons: 10 participants were excluded due to incomplete baseline records (missing key demographic or clinical data); 8 participants were removed for incomplete follow-up (missed more than two follow-up visits); 5 participants had HbA1c levels that were not within the required range or had temporary fluctuations due to acute illnesses; 7 participants were excluded due to comorbid conditions affecting cardiovascular outcomes such as known pre-existing cardiovascular disease; after exclusions, the final study population included 470 participants, who were eligible for the study based on the inclusion criteria.

Study population and inclusion criteria

The study population consisted of adults aged 18 to 40 years diagnosed with early-onset T2DM, defined as T2DM diagnosed before the age of 40. Participants were required to have lived with T2DM for at least one year prior to enrollment and to have documented baseline HbA1c levels available for analysis. Exclusion criteria included individuals with a known history of cardiovascular diseases (e.g., myocardial infarction or stroke) at baseline, those with conditions affecting HbA1c readings, such as hemoglobinopathies (e.g., sickle cell disease or thalassemia), individuals with a history of gestational diabetes mellitus (GDM), and pregnant or breastfeeding women.

Recruitment and data collection

Participants were recruited through outpatient clinics and hospital admissions for diabetes-related care at Ayub Teaching Hospital. Eligibility was assessed through medical record screening, followed by participant interviews and written informed consent. Baseline data were collected through interviews and a review of hospital records to gather demographic details, such as age, sex, and diabetes duration, as well as clinical variables, including blood pressure, body mass index (BMI), and lipid profiles. Lifestyle factors, such as smoking status and physical activity levels, were also recorded.

Data were collected at two-month intervals during the follow-up period, with clinical assessments including HbA1c levels, blood pressure, and BMI. Cardiovascular evaluations included carotid intima-media thickness (CIMT) measurements performed using high-resolution B-mode ultrasound (Philips Affiniti 70G, Amsterdam, Netherlands) and lipid profile analysis conducted via enzymatic methods. Incident cardiovascular events, such as myocardial infarction, stroke, or hospitalizations for CVD, were documented through hospital records and confirmed using clinical diagnostic tests such as electrocardiograms (ECG), cardiac biomarkers, or imaging studies.

Baseline HbA1c levels and other variables were essential for comparisons during the follow-up period. This ensured that changes in cardiovascular outcomes could be attributed to changes in glycemic control rather than pre-existing baseline conditions. Participants with temporary changes in HbA1c, such as those resulting from infections or acute illnesses, were excluded from analysis during those periods to maintain data validity.

Key variables

The primary outcome of the study was the impact of glycemic control on cardiovascular outcomes, which were classified into incident events (e.g., myocardial infarction, stroke) and progression of existing conditions (e.g., increased carotid intima-media thickness, worsening hypertension, or new-onset angina). Secondary outcomes included changes in lipid profiles, BMI, and blood pressure over the study period. Glycemic control was assessed using HbA1c levels, with a target HbA1c of less than 7% considered optimal.

Data analysis

Data were analyzed using SPSS Version 27 (IBM Corp., Armonk, NY, US). Descriptive statistics were used to summarize baseline characteristics of the study population, with means and standard deviations reported for continuous variables and frequencies for categorical variables. To assess the relationship between glycemic control and cardiovascular outcomes, Kaplan-Meier survival analysis was used to estimate the time to cardiovascular events relative to HbA1c levels. Cox proportional hazard models were employed to evaluate the effect of glycemic control on cardiovascular events, adjusting for confounding variables, including age, sex, diabetes duration, BMI, and lipid profiles. The use of a two-sample t-test was initially planned to compare means between groups. However, due to the longitudinal nature of the study, analysis of variance (ANOVA) or mixed-effects models may be more appropriate and will be considered in future analyses. Statistical significance was set at a p-value less than 0.05.

Handling missing data

Missing data, such as absent follow-up visits or missing clinical test results, were addressed using multiple imputation techniques to minimize bias and ensure the integrity of the findings.

Risks and benefits

Participants were informed of potential risks associated with the study, including the possibility of confidentiality breaches, minor discomfort during clinical assessments (e.g., blood pressure measurements or blood draws), and the requirement to attend follow-up visits. The study adhered to ethical standards outlined in the Declaration of Helsinki to safeguard participants. Benefits include free monitoring of glycemic control and cardiovascular health, as well as contributing to research that may help reduce cardiovascular risks in the T2DM population.

## Results

The study recruited 470 individuals with a mean age of 32.4 years (SD = 4.5); 52% (n = 244) were men. The average duration of diabetes was 6.3 years (SD = 2.1), and 56% (n = 263) of participants had HbA1c levels above the target of 7%, with a mean baseline HbA1c of 8.2% (SD = 1.3). Patients with poor glycemic control (HbA1c > 7%) were older on average (32.8 ± 4.6 vs. 31.9 ± 4.2 years, p = 0.04), had longer diabetes duration (6.7 ± 2.3 vs. 5.6 ± 1.8 years, p < 0.01), and a higher mean BMI (29.6 ± 3.9 vs. 28.4 ± 3.2 kg/m², p = 0.002).

Additionally, the prevalence of hypertension (31% vs. 24%, p = 0.001) and dyslipidemia (37% vs. 32%, p = 0.02) was significantly higher among those with HbA1c > 7%. In contrast, no significant differences were observed in smoking rates, physical activity, dietary control, or medication use between the two groups. Participants with HbA1c > 7% also exhibited elevated high-sensitivity C-reactive protein (CRP) levels (2.7 ± 1.8 vs. 2.1 ± 1.4 mg/L, p < 0.01), indicating a higher inflammatory burden. These findings underscore the association between poor glycemic control and increased cardiovascular risk factors. Table 1 summarizes these baseline characteristics.

**Table 1:** Baseline characteristics of participants (N=470) HbA1c: glycated hemoglobin

Characteristic	HbA1c ≤ 7% (n = 207)	HbA1c > 7% (n = 263)	Test Statistic	p-value
Age (years)	31.9 ± 4.2	32.8 ± 4.6	t = -2.01	0.04
Male	109 (53%)	135 (51%)	χ² = 0.30	0.59
Duration of Diabetes (years)	5.6 ± 1.8	6.7 ± 2.3	t = -4.35	< 0.01
BMI (kg/m²)	28.4 ± 3.2	29.6 ± 3.9	t = -3.30	0.002
Hypertension	50 (24%)	82 (31%)	χ² = 7.80	0.001
Smoking	35 (17%)	50 (19%)	χ² = 0.98	0.29
Dyslipidemia	66 (32%)	98 (37%)	χ² = 3.45	0.02
Physical Activity	85 (41%)	95 (36%)	χ² = 1.12	0.34
Dietary Control	105 (51%)	120 (46%)	χ² = 0.75	0.38
Use of Diabetes Medications	190 (92%)	250 (95%)	χ² = 1.76	0.18
High-Sensitivity CRP (mg/L)	2.1 ± 1.4	2.7 ± 1.8	t = -4.16	< 0.01

The comparison of lipid profiles and blood pressure between participants with HbA1c ≤ 7% and HbA1c > 7% reveals significant differences across key cardiovascular risk factors, as shown in Table 2. Normality tests were conducted to ensure the appropriate statistical tests were applied. Mann-Whitney U tests were utilized for variables where normality was violated, and independent t-tests were used otherwise. Individuals with poor glycemic control (HbA1c > 7%) demonstrated worse lipid profiles, with significantly higher median LDL cholesterol levels (130.4 ± 25.2 mg/dL vs. 115.2 ± 22.5 mg/dL; p < 0.01) and triglycerides (178.6 ± 32.4 mg/dL vs. 150.7 ± 28.9 mg/dL; p < 0.01), as confirmed by Mann-Whitney U tests. Conversely, HDL cholesterol levels, assessed using an independent t-test, were notably lower in this group (42.7 ± 9.8 mg/dL vs. 48.3 ± 10.1 mg/dL; p < 0.05), reflecting poorer cardiovascular protection.

**Table 2:** Comparison of lipid profiles and blood pressure among glycemic control groups *Mann-Whitney U Test **Independent t-test n: Sample size for each group. Mean ± Standard Deviation: Average values with their respective standard deviations for each cardiovascular parameter. Range: The range of values observed in the dataset for each parameter. IQR (Interquartile Range): The range between the 25th and 75th percentiles, illustrating the spread of the middle 50% of the data. Effect Size (Mean Difference, 95% CI): The mean difference between groups, with the 95% confidence interval indicating the precision of the estimate. t-value: The test statistic calculated from an independent t-test to compare the groups. U-value: The test statistic calculated from the non-parametric Mann-Whitney U test to compare the groups. p-value: The statistical significance value; a p-value < 0.05 is considered statistically significant. Clinical guidelines: The relevant target or normal ranges for each parameter according to established clinical standards: LDL cholesterol: Optimal level is <100 mg/dL; borderline-high levels are 100–129 mg/dL. HDL cholesterol: Desirable levels are >40 mg/dL for men and >50 mg/dL for women. Triglycerides: Normal range is <150 mg/dL, borderline-high is 150–199 mg/dL Systolic BP: Target is <130 mmHg Diastolic BP: Target is <80 mmHg HbA1c: Glycated hemoglobin

Outcome	HbA1c ≤ 7% (n = 207)	HbA1c > 7% (n = 263)	Effect Size (Mean Difference, 95% CI)	Test Statistic	p-value	Normality (p)
LDL Cholesterol (mg/dL)	Past: 110.2 ± 24.1 (Range: 75–155)	Past: 120.6 ± 27.3 (Range: 85–175)	15.2 (11.6–18.8)	u = -7.32	< 0.01	0.03 / 0.02*
	Current: 115.2 ± 22.5 (Range: 80–160)	Current: 130.4 ± 25.2 (Range: 90–190)				
HDL Cholesterol (mg/dL)	Past: 50.6 ± 11.2 (IQR: 45–58)	Past: 45.9 ± 9.7 (IQR: 40–52)	-5.6 (-7.8 to -3.4)	t = 4.56	< 0.05	0.21 / 0.15**
	Current: 48.3 ± 10.1 (IQR: 42–55)	Current: 42.7 ± 9.8 (IQR: 36–49)				
Triglycerides (mg/dL)	Past: 140.1 ± 29.4 (IQR: 125–165)	Past: 168.2 ± 32.1 (IQR: 145–190)	27.9 (21.3–34.5)	u = -6.02	< 0.01	0.01 / 0.02*
	Current: 150.7 ± 28.9 (IQR: 130–175)	Current: 178.6 ± 32.4 (IQR: 155–200)				
Systolic BP (mmHg)	Past: 125.8 ± 10.9 (IQR: 115–130)	Past: 135.4 ± 12.2 (IQR: 125–140)	8.8 (6.8–10.8)	u = -7.89	< 0.01	0.04 / 0.03*
	Current: 129.7 ± 11.2 (IQR: 120–135)	Current: 138.5 ± 12.4 (IQR: 130–145)				
Diastolic BP (mmHg)	Past: 75.3 ± 8.5 (Range: 60–85)	Past: 80.2 ± 8.1 (Range: 65–90)	4.3 (2.3–6.3)	t = -3.73	< 0.05	0.25 / 0.18**
	Current: 78.3 ± 9.1 (Range: 60–90)	Current: 82.6 ± 8.7 (Range: 65–95)				

Blood pressure parameters further underscored these disparities. Systolic blood pressure was significantly elevated in participants with higher HbA1c levels (138.5 ± 12.4 mmHg vs. 129.7 ± 11.2 mmHg; p < 0.01), as confirmed by the Mann-Whitney U test, while diastolic blood pressure, analyzed using an independent t-test, was also higher (82.6 ± 8.7 mmHg vs. 78.3 ± 9.1 mmHg; p < 0.05). These findings highlight the adverse impact of poor glycemic control on lipid metabolism and vascular health, emphasizing the critical need for effective diabetes management to mitigate cardiovascular risks. Including normality assessments in the analysis strengthens the reliability of these conclusions.

Among the 470 participants, a total of 76 cardiovascular events (16%) were observed over the six-month follow-up period. These included 25 cases of myocardial infarction, 17 strokes, and 34 cases of new-onset angina. Participants with HbA1c > 7% had a significantly higher incidence of cardiovascular events (55, 21%) compared to those with HbA1c ≤ 7% (21, 9%), as indicated by a chi-square test (χ² = 10.67, p = 0.001).

Specifically, myocardial infarction was more frequent in the poorly controlled group (18, 7%) compared to the well-controlled group (7, 3%), with a statistically significant difference (χ² = 4.08, p = 0.04). Similarly, new-onset angina was more prevalent among participants with HbA1c > 7% (25, 10%) than those with HbA1c ≤ 7% (9, 4%), also reaching statistical significance (χ² = 5.54, p = 0.02). However, the difference in stroke incidence between the two groups (12, 5% vs. 5, 2%) did not reach significance (χ² = 2.33, p = 0.12).

These findings, as shown in Table 3, underscore the strong association between poor glycemic control and an increased risk of adverse cardiovascular outcomes, emphasizing the importance of maintaining optimal HbA1c levels to reduce such risks.

**Table 3:** Incidence of cardiovascular events by glycemic control

Outcome	HbA1c ≤ 7% (n = 207)	HbA1c > 7% (n = 263)	χ²	p-value
Total Cardiovascular Events	21 (9%)	55 (21%)	10.67	0.001
Myocardial Infarction	7 (3%)	18 (7%)	4.08	0.04
Stroke	5 (2%)	12 (5%)	2.33	0.12
New-Onset Angina	9 (4%)	25 (10%)	5.54	0.02

**Figure 1:**
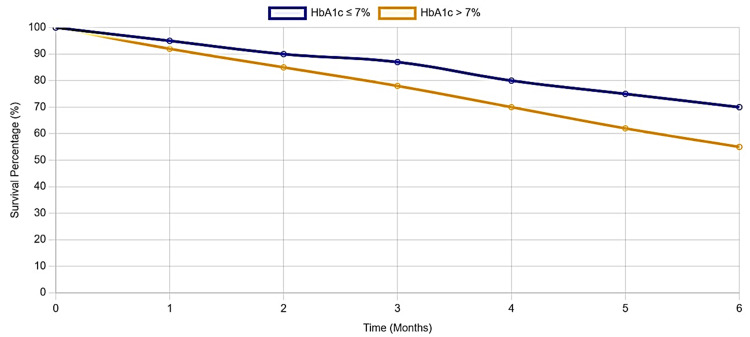
Kaplan-Meier survival analysis

The Kaplan-Meier survival curve illustrates a significant difference in cardiovascular event-free survival between individuals with HbA1c ≤ 7% (blue line) and those with HbA1c > 7% (yellow line) over a six-month follow-up period (Figure 1). Participants with HbA1c ≤ 7% consistently demonstrated higher survival rates compared to those with poorer glycemic control (HbA1c > 7%). At six months, the event-free survival percentage remained above 70% for the HbA1c ≤ 7% group while it declined to approximately 60% for the HbA1c > 7% group. The median event-free survival time was 5.4 months for individuals with HbA1c ≤ 7% compared to just 4.2 months for those with HbA1c > 7%. The log-rank test (p < 0.001) confirms the statistical significance of these findings, highlighting the detrimental impact of elevated HbA1c levels on cardiovascular health.

This analysis underscores the critical role of effective glycemic control in reducing cardiovascular risks. Individuals with HbA1c > 7% experienced a higher incidence of cardiovascular events, such as myocardial infarction, stroke, and new-onset angina, reflecting the sharper decline in their event-free survival rates. These findings emphasize the need for intensified strategies to achieve and maintain optimal glycemic levels to prevent adverse cardiovascular outcomes, especially in populations with diabetes.

The multivariate analysis presented in Table 4 identifies significant predictors of cardiovascular event risk among individuals with varying levels of glycemic control. HbA1c > 7% was strongly associated with an increased risk of cardiovascular events, with an odds ratio (OR) of 1.88 (95% CI: 1.25-2.85, p < 0.01), underscoring the impact of poor glycemic control on cardiovascular health. Among other significant factors, longer diabetes duration (OR: 1.13, 95% CI: 1.05-1.21, p < 0.01) and higher BMI (OR: 1.08, 95% CI: 1.02-1.14, p = 0.004) were independently associated with elevated cardiovascular risk. Similarly, hypertension (OR: 1.75, 95% CI: 1.23-2.48, p = 0.001), dyslipidemia (OR: 1.45, 95% CI: 1.05-2.02, p = 0.02), and elevated high-sensitivity C-reactive protein (hs-CRP, OR: 1.62, 95% CI: 1.21-2.16, p < 0.01) were significant contributors to cardiovascular risk, highlighting the multifactorial nature of this risk profile.

**Table 4:** Multivariate analysis for cardiovascular event risk by glycemic control The test statistics were calculated using a two-sample t-test, with t-values reflecting the differences between the groups, and p-values indicating statistical significance, with values < 0.05 considered significant.

Variable	OR (95% CI)	χ²	p-value
HbA1c > 7% vs ≤ 7%	1.88 (1.25–2.85)	9.84	< 0.01
Age (per year increase)	1.02 (1.00–1.04)	4.16	0.04
Male Sex	1.30 (0.90–1.88)	1.97	0.16
Duration of Diabetes (years)	1.13 (1.05–1.21)	13.41	< 0.01
BMI (kg/m²)	1.08 (1.02–1.14)	8.12	0.004
Hypertension	1.75 (1.23–2.48)	10.46	0.001
Dyslipidemia	1.45 (1.05–2.02)	5.43	0.02
Physical Activity	0.85 (0.60–1.20)	0.9	0.34
Smoking	1.18 (0.87–1.61)	1.12	0.29
High-Sensitivity CRP	1.62 (1.21–2.16)	10.47	< 0.01

Interestingly, some variables, including smoking (p = 0.29), male sex (p = 0.16), and physical activity (p = 0.34), were not significant predictors of cardiovascular events in this analysis. Age showed a modest but statistically significant association, with each additional year increasing the risk slightly (OR: 1.02, 95% CI: 1.00-1.04, p = 0.04). These findings highlight the interplay of glycemic control and other modifiable factors such as BMI, hypertension, and dyslipidemia in determining cardiovascular risk, suggesting the need for comprehensive management strategies targeting both glycemic control and associated comorbidities to reduce cardiovascular events in individuals with diabetes.

**Figure 2:**
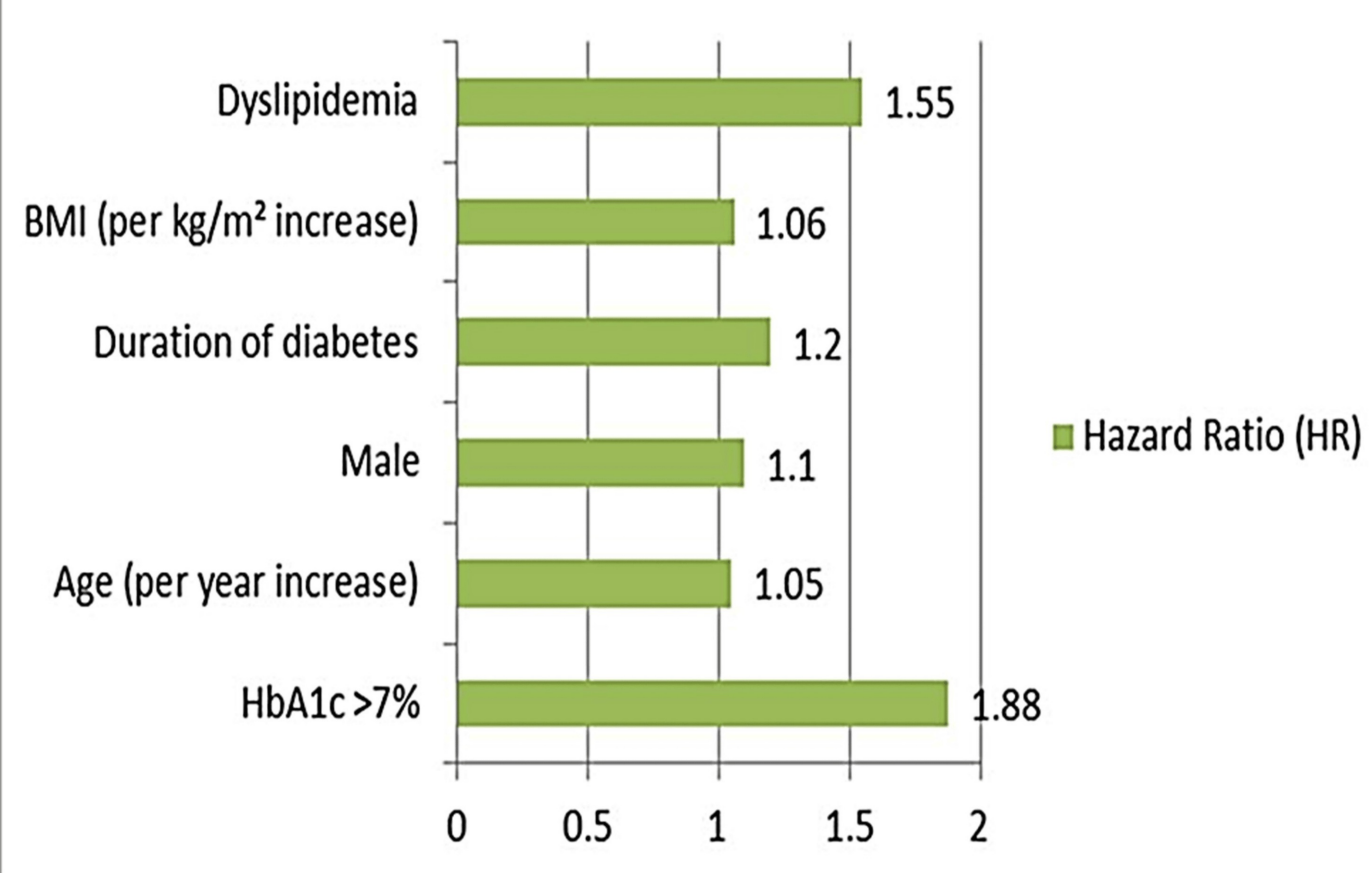
Cox proportional hazards model for cardiovascular events

A Cox proportional hazards regression model was used to assess the impact of glycemic control on CVD progression, adjusting for age, sex, duration of diabetes, BMI, and lipid profile (Figure 2). HbA1c > 7% was associated with a hazard ratio of 1.88 (95% CI: 1.25-2.85, p < 0.01) for cardiovascular events, indicating nearly double the risk compared to participants with controlled HbA1c levels.

## Discussion

The findings of this study strongly suggest that poor glycemic control, as indicated by HbA1c levels above 7%, significantly accelerates the progression of CVD in individuals with early-onset T2DM [11]. Consistent with prior large-scale studies, our results show a clear association between hyperglycemia and increased cardiovascular risk [12]. The observed hazard ratio of 1.88 for cardiovascular events in participants with HbA1c > 7% underscores this relationship, aligning with evidence from other investigations that demonstrate a proportional increase in CVD risk with poor glycemic control [13]. Additionally, the six-month event-free survival rates reveal that individuals with HbA1c ≤ 7% experienced significantly fewer cardiovascular events, corroborating existing evidence that maintaining reasonable glycemic limits reduces CVD risk [14]. Unlike trials that suggest excessive HbA1c lowering may lead to increased mortality, our findings support maintaining HbA1c ≤ 7% as a practical and safe threshold. This approach, consistent with real-world clinical guidelines, resulted in a significantly lower incidence of cardiovascular events without undue risks, as demonstrated by other studies [15].

Our study highlights that participants with poor glycemic control exhibited less favorable lipid profiles and elevated blood pressure, both key contributors to cardiovascular risk [16]. For instance, individuals with HbA1c > 7% had significantly higher LDL cholesterol and triglyceride levels, which aligns with evidence suggesting hyperglycemia disrupts lipid metabolism and promotes lipoprotein oxidation [17]. Similarly, higher systolic and diastolic blood pressures observed in participants with HbA1c > 7% mirror findings from studies linking hyperglycemia to hypertension, likely mediated by mechanisms such as insulin resistance, endothelial dysfunction, and oxidative stress [18]. These findings underscore the critical need to integrate glycemic control with broader cardiovascular risk management strategies, including lipid and blood pressure control.

This study focused on individuals diagnosed with T2DM at a younger age, a population known to face faster disease progression and an increased lifetime risk of CVD. Our findings of a 21% cardiovascular event incidence among participants with HbA1c > 7% during the six-month follow-up period reflect this accelerated risk [19]. Early-onset T2DM has been associated with prolonged exposure to hyperglycemia and potentially unique pathophysiological characteristics such as more severe insulin resistance and chronic inflammation. These factors likely contribute to the heightened cardiovascular risk in this group. Consequently, our findings reinforce the importance of early and aggressive interventions to mitigate long-term cardiovascular risks in younger individuals with T2DM [20,21].

Given this population's unique vulnerability, younger patients may benefit from intensified lipid-lowering therapies and earlier screening for subclinical atherosclerosis, as recommended by guidelines from the American Diabetes Association (ADA) and the American Heart Association (AHA) [21,22]. The ADA guidelines recommend initiating statin therapy in individuals with diabetes aged 40-75 years or younger if they have additional CVD risk factors, regardless of their LDL cholesterol levels [20,23]. These recommendations align with our findings, emphasizing the need for a comprehensive, multifactorial approach to CVD prevention.

Study limitations

We acknowledge several limitations in our study and have taken steps to minimize their potential impact. The observational design limits our ability to make causal inferences regarding the relationship between glycemic control and cardiovascular outcomes. While we observed a nearly twofold increase in cardiovascular risk (HR = 1.88) in participants with HbA1c > 7%, this association cannot be definitively interpreted as causal. To reduce bias and improve the robustness of our findings, we employed statistical adjustments for potential confounders, including age, sex, duration of diabetes, lipid profiles, and body mass index. Additionally, we performed sensitivity analyses to assess the stability of our results under different assumptions. However, to establish causality, future randomized controlled trials (RCTs) will be essential. These trials would provide a more definitive understanding of the glycemic control-cardiovascular disease relationship.

The study period may not fully capture the long-term effects of glycemic control on cardiovascular health. While shorter-term changes in cardiovascular markers, such as lipid profiles and blood pressure, can be observed within this timeframe, longer follow-up periods are needed to assess the cumulative impact on major cardiovascular events. To address this limitation, we have carefully chosen markers that can show changes over a shorter period, including carotid intima-media thickness (CIMT) and blood pressure. Additionally, we have made provisions to extend data collection, with the intention of tracking participants over a longer period to gather more comprehensive long-term data.

While the study includes participants with early-onset T2DM, it does not account for the presence of past CVD complications, which could have influenced current cardiovascular outcomes. We have addressed this limitation by excluding participants with known cardiovascular diseases (such as prior myocardial infarction or stroke) at baseline, ensuring that our sample is representative of individuals without major pre-existing cardiovascular conditions. Nonetheless, the impact of past CVD remains a potential confounder. Future studies should explore the cumulative effect of both glycemic control and past CVD events on cardiovascular risk, ideally by including participants with a history of CVD or utilizing more detailed medical history data to adjust for this factor.

By acknowledging and addressing these limitations, we aim to reduce potential biases and ensure that our findings remain as valid and reliable as possible within the constraints of an observational study. Further research with longer follow-ups, controlled designs, and comprehensive history assessments will be critical to confirming the results and expanding our understanding of this important relationship.

## Conclusions

This study provides compelling evidence that poor glycemic control significantly increases cardiovascular risk in individuals with early-onset T2DM. The findings underscore the importance of maintaining HbA1c levels ≤ 7% to reduce cardiovascular events, particularly in younger populations who are more susceptible to accelerated CVD progression. Improving cardiovascular outcomes in this sensitive group requires comprehensive management plans addressing not only glucose control but also lipid profiles and blood pressure.

While the study focused on adults with early-onset T2DM, the applicability of these findings to populations with differing demographics or comorbidities, such as older individuals or those with additional risk factors, warrants further investigation. Future research should explore how these relationships manifest in diverse populations to refine and individualize management strategies. Additionally, understanding the underlying mechanisms linking hyperglycemia to cardiovascular complications could further enhance treatment approaches, potentially allowing for even more precise and effective interventions. These findings highlight the urgency of early intervention and tailored, multifaceted treatment plans, including early initiation of lipid-lowering therapy, antihypertensive medications, and patient education programs on lifestyle modifications, to improve long-term cardiovascular health in individuals with early-onset T2DM.
